# GOBeacon: An ensemble model for protein function prediction enhanced by contrastive learning

**DOI:** 10.1002/pro.70182

**Published:** 2025-06-22

**Authors:** Weining Lin, David Miller, Zhonghui Gu, Christine Orengo

**Affiliations:** ^1^ Institute of Structural and Molecular Biology University College London London UK; ^2^ Centre for Artificial Intelligence University College London London UK; ^3^ Peking‐Tsinghua Center for Life Sciences Peking University Beijing China

**Keywords:** contrastive learning, protein function prediction, protein interaction network, protein language model

## Abstract

Accurate prediction of protein function is fundamental to understanding biological processes, with computational methods becoming increasingly essential as experimental methods struggle to keep pace with the rate of newly discovered proteins. Despite significant advances in machine learning approaches, existing methods often fail to capture the complex relationships between protein structure, evolution, and function, leading to limited prediction accuracy. The challenge lies in effectively integrating diverse biological data types while maintaining computational efficiency. Here, we show that GOBeacon, a novel ensemble model integrating structure‐aware protein language model embeddings with protein–protein interaction networks, achieves high accuracy in protein function prediction. By employing a contrastive learning framework, GOBeacon demonstrates superior performance on the sequence‐based CAFA3 benchmark, achieving *F*_max_ scores of 0.561 (BP), 0.583 (MF), and 0.651 (CC), outperforming existing methods including domain‐PFP and DeepGOPlus. The model's effectiveness extends to structure‐based function prediction tasks, where it matches or exceeds the performance of specialized structure‐based tools like HEAL and DeepFRI, while not being explicitly trained on structure. We anticipate that GOBeacon's architecture will serve as a foundation for next‐generation protein analysis tools, while its modular design enables future integration of additional data types and improved prediction capabilities. These advances represent a significant step toward reliable automated protein function annotation, addressing a critical bottleneck in modern biology. GOBeacon is now publicly available: https://github.com/wlin16/GOBeacon.git

## 1 INTRODUCTION

Proteins are crucial in managing the diverse biological functions necessary for cellular life, including key molecular processes such as cell signaling, immune responses, and various aspects of cellular metabolism. The advent of high‐throughput DNA sequencing has led to exponential growth in the collection of protein sequences, now far outpacing the capacity for experimental functional annotation. For example, the Big Fantastic Database currently contains 2.5 billion sequences (Jumper et al., 2021). Traditionally, scientists have relied on slow, labor‐intensive, and costly low‐throughput wet lab experiments to determine protein functions. This approach has resulted in most protein sequences lacking functional annotations (Radulovic et al., 2013; Zhou et al., 2019). Although broad annotations, such as classifying enzyme families, can often be inferred through sequence homology and protein domain motifs, the complex task of identifying more fine‐grained molecular functions requires detailed experimental characterization.

Traditional bioinformatic methods attempt to infer protein function primarily using sequence alignment techniques. These techniques, such as identifying similar domains via Pfam (Mistry et al., 2021) or using local alignments such as BLAST (Altschul et al., 1990; Buchfink et al., 2015), enable the transfer of functions from previously experimentally validated proteins to new sequences. More recently, advances in machine learning have led to promising computational approaches for predicting protein functions (Fa et al., 2018; Gelman et al., 2021; Kulmanov et al., 2018; Yang et al., 2015). Notably, in the Critical Assessment of Functional Annotation (CAFA) challenge, which focuses on blind predictions, machine learning methodologies have surpassed traditional sequence alignment‐based methods in accuracy (Radulovic et al., 2013). Existing machine learning‐based methods for protein function prediction can be broadly classified into three categories: sequence‐based, structure‐based, and protein interaction‐based approaches.

For sequence‐based methods, recently published models such as DeepGOPlus and ATGO, employing convolutional or Transformer‐based neural networks (Kulmanov et al., 2018; Zhu et al., 2022) leverage detailed protein sequence representations, that is, embeddings, that capture rich patterns within the data. These sequence‐embeddings‐based methods report much higher prediction accuracy compared to traditional alignment methods mentioned above. Furthermore, methods such as DomainPFP (Ibtehaz et al., 2023) improve the precision of function prediction by integrating domain‐specific features and protein function annotation representation during model training, which helps transfer functional annotations from well‐characterized domains to novel sequences. However, since protein structure is more conserved than sequence, the high sequence diversity among functionally similar proteins limits sequence‐based methods, driving interest in structural approaches.

Structure‐based methods have emerged as an effective approach by leveraging the fundamental relationship between protein 3D structure and function (Gligorijević et al., 2021; Lai & Xu, 2022; Zhao et al., 2022). Recent advances in protein structure prediction (Baek et al., 2021; Jumper et al., 2021) have made protein contact maps and 3D structures more accessible. These developments have facilitated the use of deep learning structure‐based methods, which often model 3D structures as graphs and use graph neural network (GNN) architectures (Kipf & Welling, 2017), employing the message passing paradigm (Gilmer et al., 2017), to process structural data (Gligorijević et al., 2021; Gu et al., 2023). For example, DeepFRI encodes amino acids as graph nodes, connecting them with edges if they are less than 10 Å apart, and applies a graph convolutional neural network to learn complex structure–function relationships. This approach captures spatial relationships more effectively than sequence‐based methods, enhancing function prediction accuracy. Despite progress, challenges persist in using structural information to predict protein functions. Deep learning structure methods face GPU memory constraints with large proteins, while the limitations of shallow GNNs, particularly their tendency to over‐smooth features, make it difficult to capture long‐range structural relationships and identify key residues. These limitations have motivated researchers to explore complementary approaches that incorporate additional biological data.

Additional approaches explore protein function prediction using data from protein–protein interaction (PPI) networks (Cho et al., 2016; Mostafavi et al., 2008; You et al., 2021). These methods rely on the principle that interacting proteins often share similar functions or participate in related pathways, offering valuable insights for enhancing prediction accuracy (Oliver, 2000; Schwikowski et al., 2000). DeepGraphGO exemplifies this approach by combining protein sequence and network information through GNNs, reaching state‐of‐the‐art (SOTA) performance (You et al., 2021). However, these techniques face limitations due to the quality and completeness of interaction data, affecting prediction accuracy.

**FIGURE 1:**
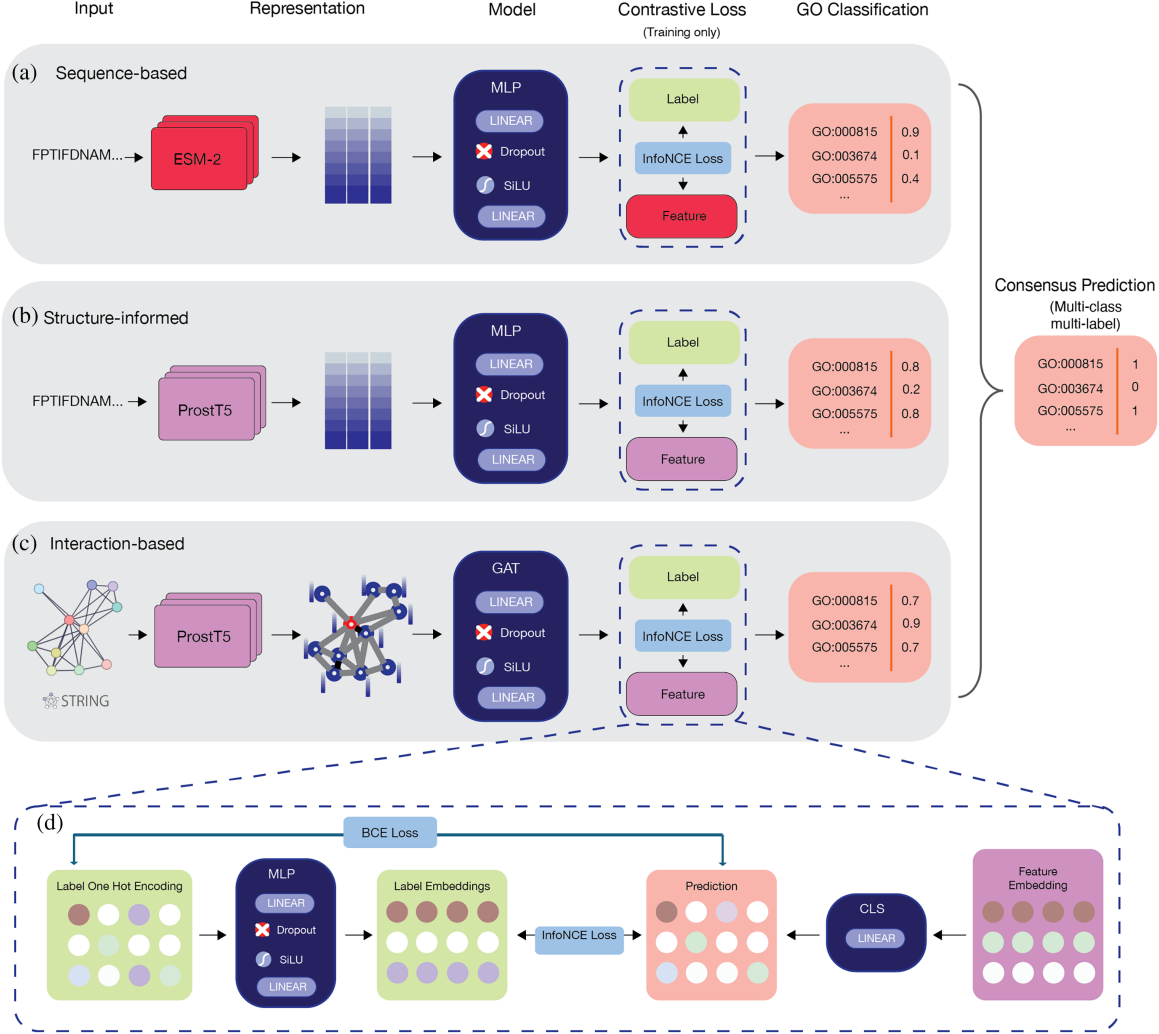
Overview of GOBeacon. GOBeacon predicts protein function using three models: (a) Sequence‐based: Protein sequences are embedded with ESM‐2, then processed by a multi‐layer perceptron (MLP) featuring linear, SiLU activation, dropout, and multi‐class output layers. (b) Structure‐informed: Sequences are embedded using ProstT5, followed by an identical MLP architecture. (c) Interaction‐based: Protein interaction networks (from STRING) are represented as graphs using ProstT5 node embeddings, processed by a graph attention network (GAT) with a multi‐class output. (d) During training, all models utilize InfoNCE contrastive loss, comparing true labels with learned feature representations to enhance prediction accuracy. Final predictions are generated by averaging the outputs of all three models to determine protein function. CLS: Classification layer.

Here, we introduce GOBeacon (Figure 1), an ensemble model for protein function prediction combining three critical modalities: protein sequences, protein structures, and interaction information. For the sequence‐based method, we utilized embeddings of ESM‐2, a protein language model (PLM) trained on 250 million protein sequences (Lin et al., 2023). For the structure‐informed method, rather than training directly on 3D protein structures as inputs, we employed ProstT5 (Heinzinger et al., 2024), a PLM pre‐trained to translate between protein sequence and their corresponding structures in 3D‐alphabet format, introduced by Foldseek (Van Kempen et al., 2023). This approach leverages the structural data implicit in the training of ProstT5 to enhance our model's predictive capabilities without the need for explicit structural inputs during our analysis. For the interaction‐based method, we constructed a PPI graph using data from the STRING database and used ProstT5 embeddings as node features. By integrating embeddings from learned evolutionary representations via ESM‐2, structure‐aware representations from ProstT5, and interaction networks, GOBeacon achieves high predictive accuracy in predicting protein functions across diverse conditions.

Additionally, we incorporate contrastive learning to optimize the model's performance. This strategy enhances the model by minimizing the distance between an anchor and a positive sample while maximizing the distance between the anchor and a negative sample. This contrastive objective is applied as a regularization term alongside supervised learning to optimize the model's performance (Heinzinger et al., 2022; Khosla et al., 2020). This approach has been shown to improve the performance in enzyme function prediction by optimizing the functional similarities among proteins (Yu et al., 2023).

To assess our model, we benchmarked GOBeacon against established methods including sequence‐based BLAST (Altschul et al., 1990), Domain‐PFP (Ibtehaz et al., 2023), DeepGOPlus (Kulmanov & Hoehndorf, 2021), PhiGnet (Jang et al., 2024), and MIF2GO (Ma et al., 2024) as well as structure‐based HEAL (Gu et al., 2023), DeepFRI (Gligorijević et al., 2021), and DPFunc (Wang et al., 2025). The benchmark datasets used for this comparison were the CAFA3 for sequence‐based methods and PDBch for the structure‐based methods.

## 2 RESULTS

### 2.1 Model investigation and optimization

#### 2.1.1 Evaluating graph neural network architectures

**FIGURE 2:**
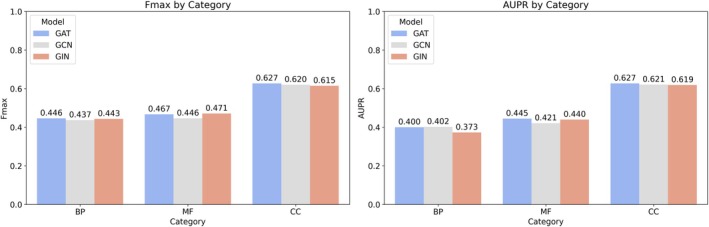
The comparison among three different graph neural network architectures, GAT, GCN, and GIN, for the interaction‐based method. The bar charts display the *F*_max_ and area under the precision‐recall curve (AUPR) across three categories: BP, MF, and CC.

A comparative analysis of different GNN architectures was conducted, focusing on graph isomorphism network (GIN), graph convolutional network (GCN), and graph attention network (GAT), with results presented in Figure 2. Performance was assessed using the *F*_max_ and AUPR metrics across the three GO sub‐ontologies: BP, MF, and CC. In the BP category, GAT achieved an *F*_max_ score of 0.446, closely followed by GCN at 0.437 and GIN at 0.443. For the MF category, GAT exhibited a slightly improved *F*_max_ of 0.467 compared to GCN's 0.446, with GIN following at 0.471. Notably, in the CC category, GAT has the strongest performance, achieving an *F*_max_ of 0.627, while GCN and GIN scored 0.620 and 0.615, respectively.

Given GAT's overall performance across all categories and metrics, it was selected as the graph architecture for the interaction‐based method in the ensemble model. Nevertheless, the observed strengths of each architecture, particularly GIN's performance in MF, highlight the potential benefits of considering the specific functional category when selecting an optimal GNN architecture for future protein function prediction tasks.

### 2.2 Performance gains from contrastive learning

**FIGURE 3:**
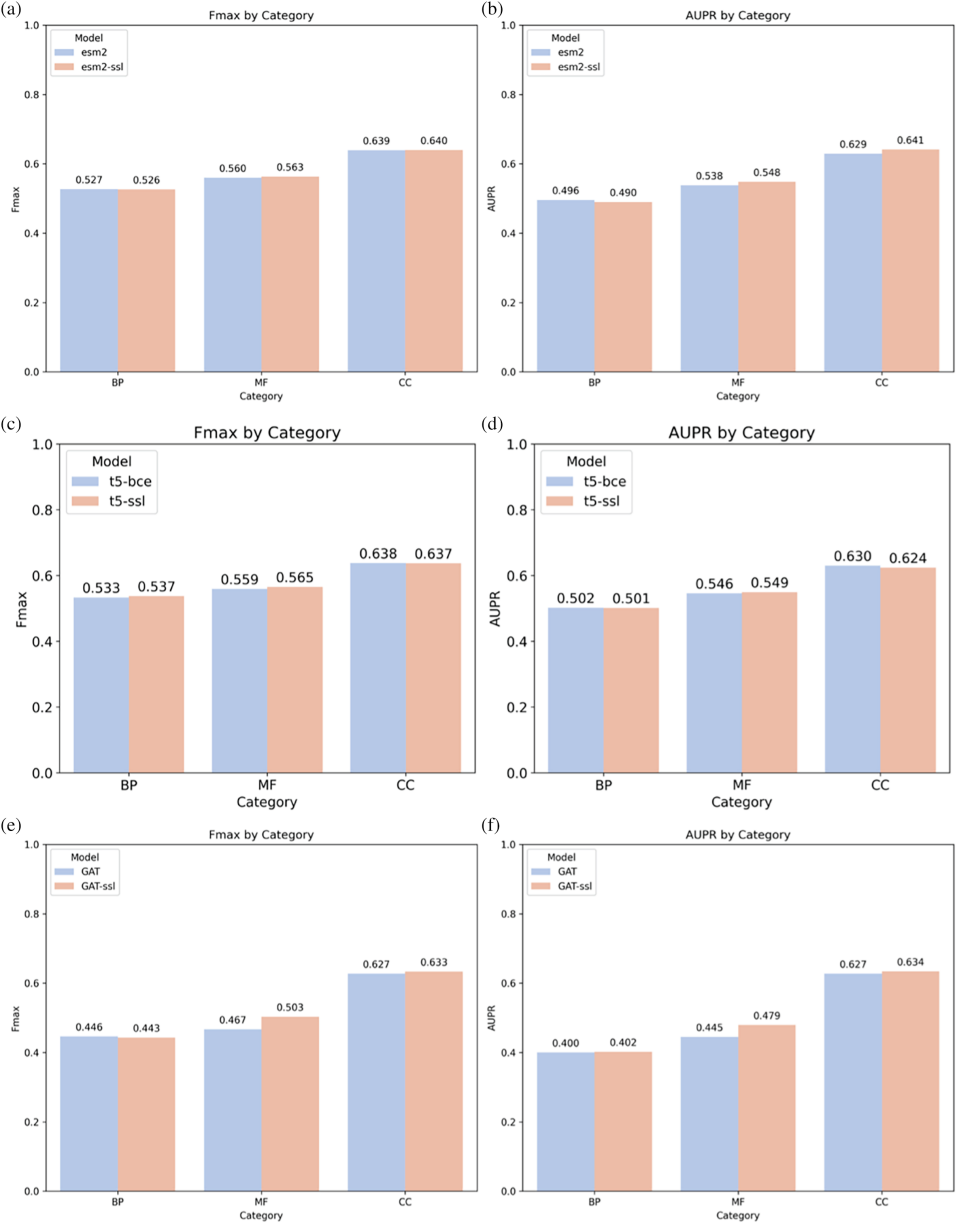
Comparative performance of loss functions across models trained independently by three modalities. This figure illustrates the performance of models trained with binary cross‐entropy and contrastive learning across three distinct model modalities: Sequence‐based (ESM‐2 embeddings), structure‐informed (ProstT5 embeddings), and interaction‐based (graph attention network, GAT). For each modality, we compare models trained using binary cross‐entropy (esm2, t5‐bce, GAT) against those trained with contrastive learning (esm2‐ssl, t5‐ssl, GAT‐ssl). Performance is evaluated using *F*_max_ (left panels) and AUPR (right panels) metrics for the three sub‐ontologies: BP, MF, and CC. The figure allows for a direct comparison of the efficacy of binary cross‐entropy and contrastive learning loss functions in enhancing the performance of function prediction models across different input data types.

The integration of contrastive learning was shown to enhance the performance of models trained on three distinct modalities, particularly in the MF and CC sub‐ontologies, as illustrated in Figure 3. For the sequence‐based models with ESM‐2 embeddings (Figure 3a, b), using a self‐supervised contrastive learning loss (esm2‐ssl) led to a notable *F*_max_ increase in the MF category (0.563 vs. 0.560) and the CC category (0.640 vs. 0.639). When applied to the structure‐informed method (Figure 3c, d), using ProstT5 embeddings with a self‐supervised learning loss (t5‐ssl) resulted in a marginal *F*_max_ improvement in the BP category (0.537 vs. 0.533) and a more substantial gain in the MF category (0.565 vs. 0.559).

The most pronounced benefits of contrastive learning were observed in the interaction‐based models using the GAT architecture (Figure 3e, f). Here, self‐supervised contrastive learning (GAT‐ssl) significantly boosted both *F*_max_ and AUPR scores, particularly in the MF (*F*_max_: 0.503 vs. 0.467, AUPR: 0.479 vs. 0.445) and CC (*F*_max_: 0.633 vs. 0.627, AUPR: 0.634 vs. 0.627) categories. While the BP category showed only marginal improvements, the overall trend across all modalities and sub‐ontologies demonstrates the effectiveness of using contrastive learning in enhancing model performance. These findings underscore the potential of employing contrastive learning techniques to boost the predictive capabilities of models in protein function prediction tasks.

### 2.3 Impact of interaction data on model performance

To investigate the impact of interaction data quality on model performance, we explored the use of more stringent criteria for defining PPIs. The STRING database provides confidence scores for each interaction pair, reflecting the nature and quality of supporting evidence. While our initial approach utilized the top 100 interactions per protein based on these scores, we hypothesized that restricting the interaction graph to higher‐confidence interactions might improve model accuracy. Therefore, we reconstructed the interaction graphs to include only interactions with a confidence score exceeding 0.7, effectively filtering for interactions deemed more reliable.

However, contrary to our hypothesis, models trained with the same GAT‐ssl architecture and hyperparameters on these restricted graphs demonstrated a decline in performance. Specifically, the *F*_max_ remained unchanged at 0.443 in the BP category (from 0.446), decreased from 0.503 to 0.474 in the MF category, and dropped from 0.633 to 0.622 in the CC category. This suggests that while higher‐confidence interactions are individually more reliable, their limited number may result in a less comprehensive representation of the underlying biological networks. Consequently, the model may lose the ability to generalize across diverse protein functions due to an overly narrow view of the PPI landscape. This underscores the limitations of relying solely on high‐confidence interactions, suggesting that a balance between interaction confidence and the diversity of the interaction network is important for model performance in protein function prediction.

### 2.4 Ensemble model performance

To effectively leverage the complementary information encoded within evolutionary data, protein structures, and PPIs, we developed GOBeacon, an ensemble model that integrates these diverse modalities. GOBeacon combines the predictive power of three distinct, contrastive‐learning enhanced models: a structure‐informed model based on ProstT5 embeddings (t5‐ssl), a sequence‐based model using ESM‐2 embeddings (esm2‐ssl), and an interaction‐based model built upon a Graph Attention Network (GAT‐ssl). Each model contributes equally to the final prediction, capitalizing on the unique strengths of its respective data type.

**FIGURE 4:**
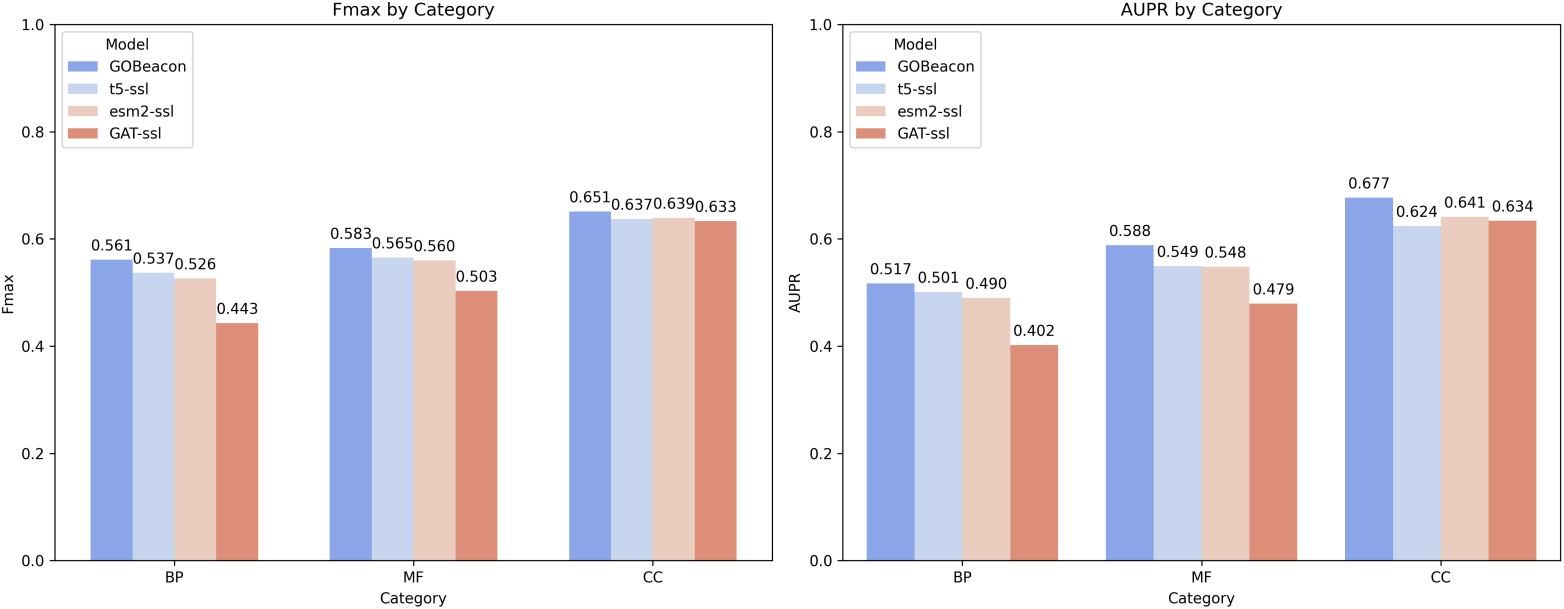
Comparison between the models trained on individual data types with the ensemble model GOBeacon across three categories on the CAFA3 benchmark.

Evaluation of the GOBeacon ensemble model on the CAFA3 benchmark (Figure 4) revealed significant performance gains across all GO sub‐ontologies relative to models trained with a single modality (i.e., models based solely on sequence, structure, or graph data). Specifically, in the BP category, GOBeacon achieved an *F*_max_ of 0.561 and an AUPR of 0.517, outperforming each individual model. In the MF category, it reached an *F*_max_ of 0.583 and an AUPR of 0.588, while the most pronounced improvements were observed in the CC category, where the ensemble attained an *F*_max_ of 0.651 and an AUPR of 0.677. These results underscore that the ensemble approach effectively leverages complementary information from diverse data modalities, resulting in significant enhancements in protein function prediction compared to models utilizing a single modality.

## 3 GOBeacon IMPROVES PROTEIN FUNCTION PREDICTION

### 3.1 CAFA3 sequence‐based benchmark

**FIGURE 5:**
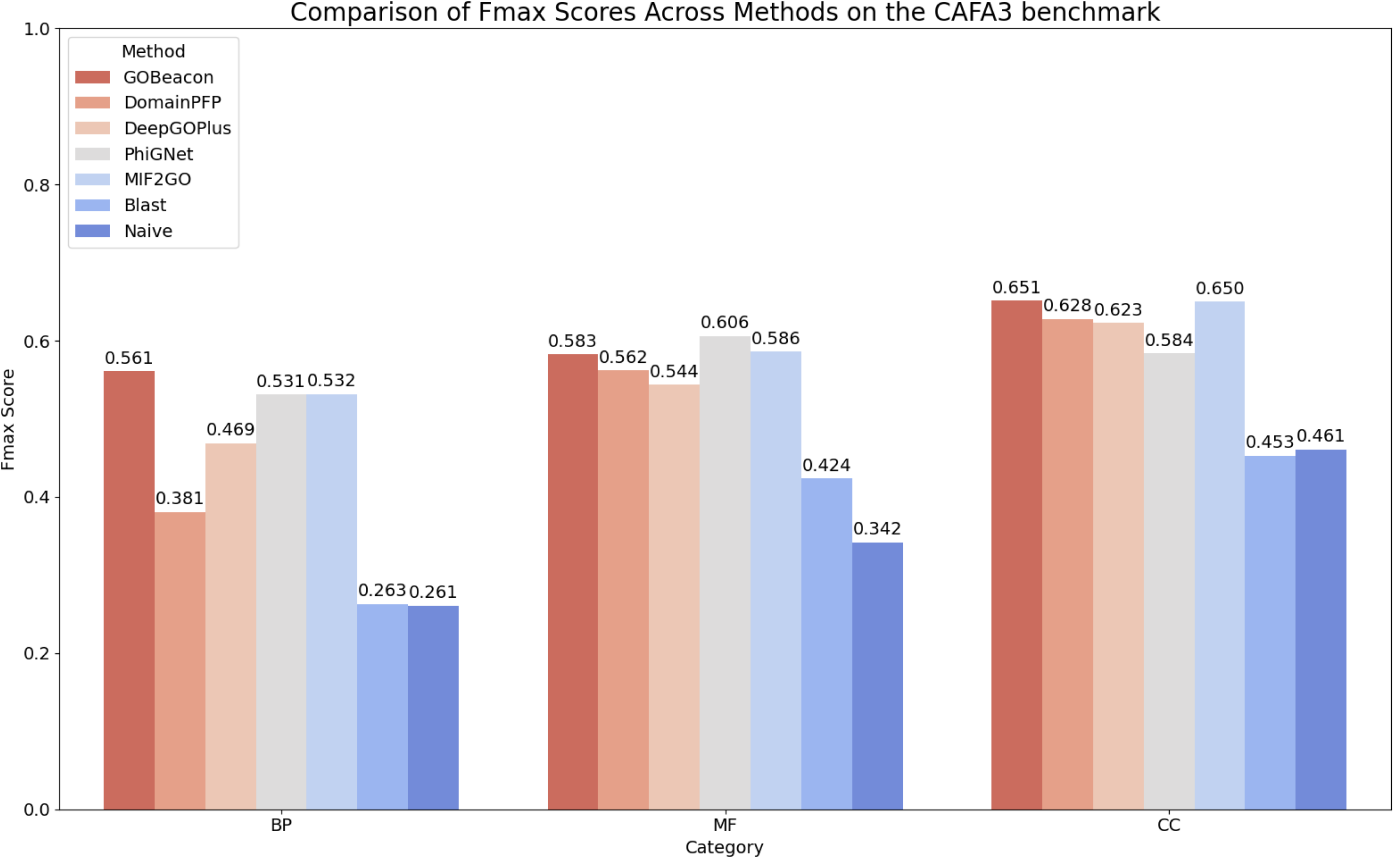
Comparison of Fmax scores across different methods on the CAFA3 benchmark. GOBeacon performance was compared against six state‐of‐the‐art methods (DomainPFP, DeepGOPlus, PhiGNet, MIF2GO, Blast, and Naive) in three gene ontology (GO) sub‐ontologies: biological process (BP), molecular function (MF), and cellular component (CC). GOBeacon achieved the highest overall average Fmax score, performing best in BP and CC, and competitively in MF.

To compare to sequence‐based methods, we trained GOBeacon using the training set provided by the CAFA3 challenge organizers and benchmarked against several SOTA methods on the CAFA3 test set, including DeepGOPlus, DomainPFP, PhiGNet, MIF2GO, BLAST, and a Naive (label frequency) method, across the three GO sub‐ontologies using the *F*_max_ metric (Figure 5). On the CAFA3 benchmark, GOBeacon achieved the highest overall average *F*_max_ score among all methods, demonstrating superior performance in BP (0.561) and CC (0.651), and competitive performance in MF (0.583). Specifically, in BP, GOBeacon outperformed DeepGOPlus (0.469), PhiGNet (0.531), MIF2GO (0.532), DomainPFP (0.381), and notably exceeded both BLAST (0.263) and the Naive method (0.261). In MF, PhiGNet obtained the highest score (0.606), followed by MIF2GO (0.586) and GOBeacon (0.583), which still surpassed DomainPFP (0.562), DeepGOPlus (0.544), BLAST (0.424), and Naive (0.342). In CC, GOBeacon achieved the best performance (0.651), marginally ahead of MIF2GO (0.650), DomainPFP (0.628), DeepGOPlus (0.623), PhiGNet (0.584), Naive (0.461), and BLAST (0.453) (Table 1).

**TABLE 1:** Comparison of *F*_max_ with 7 methods on the CAFA3 benchmark.

Model	BP	MF	CC	Avg.
GOBeacon	**0.561**	0.583	**0.651**	**0.598**
DomainPFP	0.381	0.562	0.628	0.525
DeepGOPlus	0.469	0.544	0.623	0.545
PhiGNet	0.531	**0.606**	0.584	0.573
MIF2GO	0.532	0.586	0.650	0.589
BLAST	0.263	0.424	0.453	0.380
Naive	0.261	0.342	0.461	0.355

*Note:* Bold indicates highest value in each column and underline indicates the second‐highest value in each column.

### 3.2 PDBch structure‐based benchmark

**FIGURE 6:**
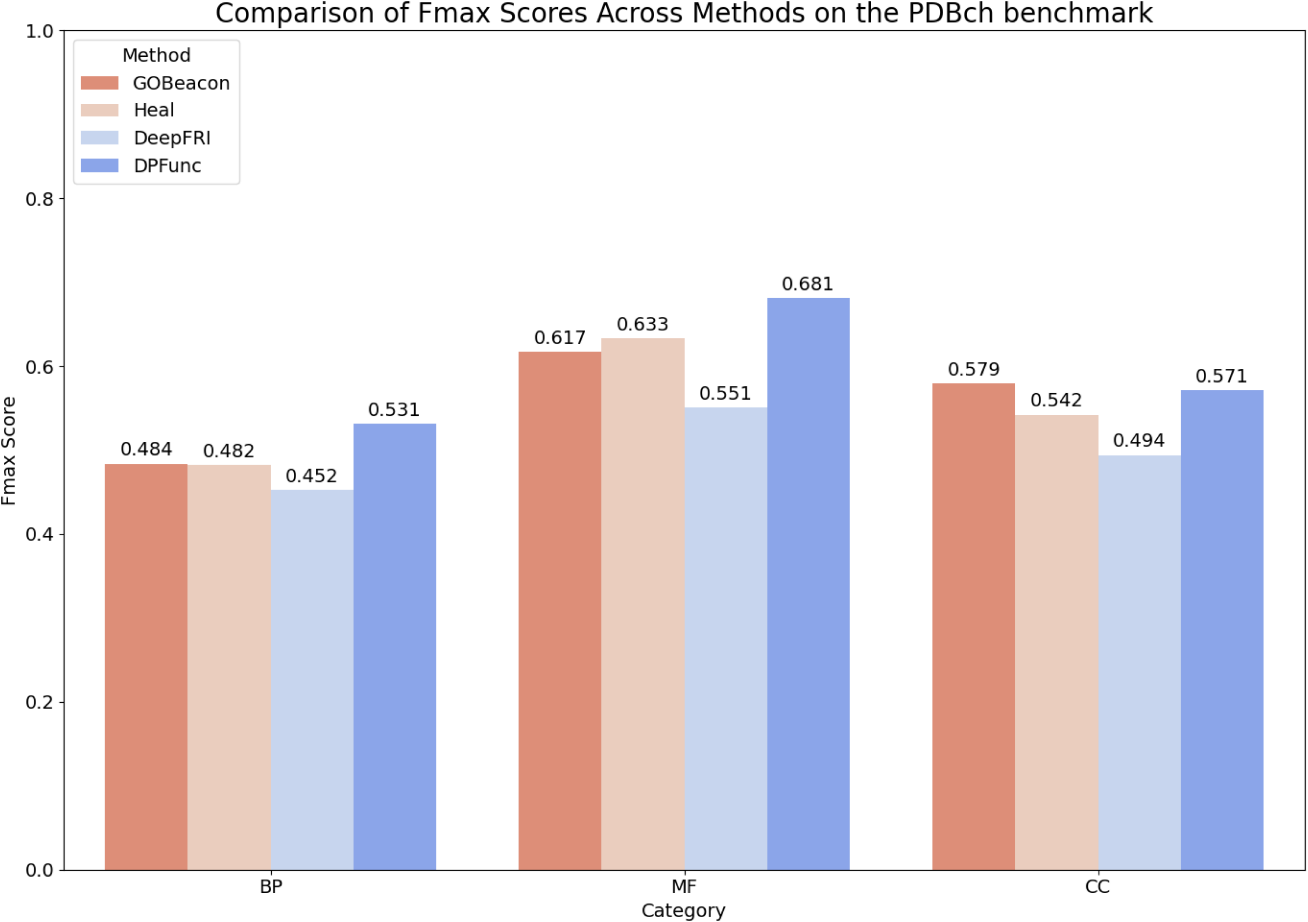
Comparison of Fmax scores across different methods on the PDBch benchmark. GOBeacon performance was evaluated against three structure‐based methods (HEAL, DeepFRI, and DPFunc) across three gene ontology (GO) sub‐ontologies: biological process (BP), molecular function (MF), and cellular component (CC). DPFunc achieved the highest average performance overall, likely due to its use of domain‐guided structure information. GOBeacon showed strong performance, particularly in the CC category, possibly benefiting from incorporating protein–protein interaction data.

For a valid comparison with structure‐based methods, GOBeacon was retrained using the sequences derived from PDB structures in the PDBCh training set and evaluated on the associated test set. When evaluated on the PDBCh benchmark, GOBeacon demonstrates competitive performance compared to structure‐based methods such as HEAL, DeepFRI, and DPFunc, even without explicitly being trained on three‐dimensional protein structures (Table 2, Figure 6). In the BP category, GOBeacon achieved an *F*_max_ of 0.484, similar to HEAL (0.483), outperforming DeepFRI (0.452), but slightly below DPFunc (0.531). In the MF category, GOBeacon (0.617) closely approached HEAL (0.634), outperformed DeepFRI (0.551), yet was behind DPFunc (0.681). Notably, in the CC category, GOBeacon's Fmax score of 0.579 exceeded HEAL (0.542) and DeepFRI (0.494), and was slightly higher than DPFunc (0.571). Overall, DPFunc achieved the highest average Fmax score (0.594), with GOBeacon following closely behind (0.560). We hypothesize that GOBeacon's strong performance, particularly in predicting protein functions within the Cellular Component category, may partly result from incorporating additional PPI data, providing an advantage over models trained solely on protein structures. However, DPFunc achieves even better overall performance, potentially due to its incorporation of domain information. This domain‐guided approach appears to significantly enhance DPFunc's ability to capture important functional residues and structural motifs, resulting in improved predictive accuracy.

**TABLE 2:** Comparison of *F*_max_ with four methods on the PDBch benchmark.

Model	BP	MF	CC	Avg.
GOBeacon	0.484	0.617	**0.579**	0.560
HEAL	0.483	0.634	0.542	0.553
DeepFRI	0.452	0.551	0.494	0.499
DPFunc	**0.531**	**0.681**	0.571	**0.594**

## 4 DISCUSSION

This study introduces GOBeacon, a novel ensemble model for protein function prediction that leverages contrastive learning and integrates three critical data modalities: evolutionary information, protein structure, and PPIs. Central to GOBeacon's success is its strategic use of PLM embeddings as rich feature representations. Recognizing that accurate protein function prediction necessitates a comprehensive approach, GOBeacon departs from traditional single‐modality methods by combining the strengths of three distinct models, each trained on one of these modalities and each empowered by the sophisticated representations derived from protein language models. This ensemble strategy, fueled by information‐rich embeddings, allows GOBeacon to capitalize on the complementary information encoded within each data source, leading to more accurate and robust predictions.

A key finding of our study is the superior performance of structure‐informed models based on ProstT5 embeddings. Even when used independently, these models outperformed those based solely on sequence or interaction data across the CAFA3 benchmark. Notably, statistical tests confirmed that incorporating contrastive (self‐supervised) learning significantly enhanced performance compared to standard BCE‐based models across all three GO sub‐ontologies (BP, MF, and CC), highlighting the general effectiveness of contrastive learning in refining already powerful embeddings. Furthermore, while the graph‐only model achieved the lowest individual performance, our analysis demonstrated that the addition of graph information significantly improved performance, indicating the complementary value of PPI networks. Ultimately, GOBeacon, by integrating sequence, structure, and graph‐based interaction data, achieved the highest overall accuracy, underscoring the synergistic effect of combining diverse data modalities.

Building upon GOBeacon's current strengths, we envision several promising avenues for future development. One direction involves optimizing the use of graph information. Our findings highlight the limitations of relying solely on high‐confidence interactions, suggesting the need for more nuanced approaches that consider the broader network topology, potentially including more distant relational layers. A second direction focuses on enhancing contrastive learning by leveraging the hierarchical structure of GO. This could involve treating proteins within the same GO branch as positive examples and those from different branches as negatives, thereby improving the model's ability to discern biologically relevant relationships. Furthermore, incorporating richer contextual information, such as GO term semantics or the comprehensive protein representations offered by the recently released ESM3 (Hayes et al., 2024), could significantly enhance GOBeacon's understanding of protein function. Additionally, our experiments on the structure‐based PDBch benchmark highlighted the superior performance of DPFunc, underscoring the continued importance of incorporating 3D structural information. Specifically, domain‐guided structural features were highly beneficial in accurately capturing key functional residues and structural motifs relevant to protein functions. Hence, in future iterations of GOBeacon, we plan to integrate more extensive 3D structural information and domain‐specific features to further enhance predictive accuracy and biological interpretability. These strategies, aimed at both refining existing components and integrating new information sources, hold the potential to further elevate GOBeacon's performance and solidify its position as a leading tool for protein function prediction.

GOBeacon represents a significant advancement in protein function prediction. Its ensemble architecture, combined with the strategic use of contrastive learning and information‐rich PLM embeddings, demonstrates the power of integrative approaches. GOBeacon not only establishes a new benchmark for accuracy but also provides a simple framework for future development, paving the way for more reliable and comprehensive protein function annotation systems.

## 5 METHODS

### 5.1 An overview of GOBeacon

GOBeacon is an ensemble model that integrates three distinct, independently trained models, each specialized to process a key data modality (Figure 1). The first model is a sequence‐based model, trained on embeddings extracted from ESM‐2 (Lin et al., 2023), a PLM rich with evolutionary information. The second model is a structure‐informed model that utilizes structure‐aware embeddings from ProstT5 (Heinzinger et al., 2024). The third model employs an interaction‐based approach, incorporating network information derived from PPIs in the STRING database.

#### 5.1.1 Sequence‐based model

The sequence‐based component of GOBeacon is designed to analyze protein sequence data by leveraging evolutionary insights embedded within the sequences. The model utilizes ESM‐2 (Lin et al., 2023), a PLM trained on a vast dataset of 250 million protein sequences. This extensive training enables ESM‐2 to recognize complex evolutionary patterns and derive rich information from protein sequences. ESM‐2 has demonstrated remarkable utility in various downstream tasks, including fitness prediction and protein design (Notin et al., 2023; Wittmann et al., 2021). highlighting the wealth of evolutionary information captured in its embeddings. This makes it highly effective for predicting protein functions. For a detailed description of the sequence‐based model training, please refer to the Supplementary Material—Methods—Sequence‐based model.

#### 5.1.2 Structure‐informed model

Although the input to the structure‐informed model is limited to primary protein sequences, it leverages a recently released PLM named ProstT5. ProstT5 was trained not only on a large dataset of protein sequences but also on protein structures using the 3Di‐alphabet format introduced by the 3D comparison tool Foldseek (Van Kempen et al., 2023). This allows ProstT5 to learn the relationship between sequence and structure. The embeddings produced by ProstT5 have shown an enhanced ability to capture higher‐level structural information. Therefore, utilizing the structure‐aware embeddings from ProstT5 is expected to be highly effective for predicting protein functions, as structure ultimately constrains function. For a detailed description of the structure‐informed model training, please refer to the Supplementary Material—Methods—Structure‐informed model.

#### 5.1.3 Interaction‐based model

The interaction‐based model within GOBeacon is designed to analyze PPI network data sourced from the STRING database version 11.5 (Szklarczyk et al., 2023). To construct the interaction network for each query protein, the top 100 neighboring proteins are selected based on the confidence scores of their connecting edges. In this network, each protein is represented as a node, and interactions between proteins are depicted as edges. Node features are derived from pooled ProstT5 embeddings. For proteins lacking neighboring nodes (i.e., no direct interactions are identified), the model forms a self‐loop to ensure their inclusion in the network analysis.

To determine the most effective GNN architecture for protein function prediction, three architectures were evaluated: GCN, GIN, and GAT. Each model offers unique advantages for handling the complexities of protein function prediction within the network framework. Given GAT's superior performance over GCN and GIN, it was chosen as the model architecture for the graph‐based method. Details on the selection of the GNN and the construction of the interaction‐based methods are available in the Supplementary Material—Methods—Interaction‐based model.

#### 5.1.4 Optimization by contrastive learning

In this study, contrastive learning is central to enhancing the model's ability to detect functional similarities among proteins by incorporating a dual‐loss framework that combines a supervised prediction loss with a contrastive InfoNCE loss (He et al., 2020) augmented by a label embedding alignment module. Drawing inspiration from previous work that demonstrated how a contrastive learning approach could significantly boost protein function prediction accuracy (Fu et al., 2024), we adapted and extended this strategy. In our approach, the label embeddings are generated by passing the protein's label vector, which is one‐hot encoded, through a network composed of two linear layers with an activation function in between (pseudocode Algorithm 1). The supervised loss directly compares protein feature outputs with one‐hot encoded labels to ensure precise classification, while the contrastive loss transforms these one‐hot labels into rich, continuous embeddings that capture context‐aware semantic relationships, subsequently aligning them with protein embeddings in the hidden space by treating each protein's own label as the positive sample and all other labels as negatives, thereby encouraging each protein's representation to be close to its corresponding label embedding and distant from the embeddings of other labels, as detailed in Equation (1). This approach enables the model to learn the intricate relationships among labels, ultimately optimizing both categorical accuracy and semantic structure for improved functional recognition. Importantly, the contrastive loss is employed only during training; during the testing and inference stage, the model uses the pretrained embedding and projection layers, without any contrastive loss or label input, to generate its final function predictions.

ALGORITHM 1 **Loss computing**

**def compute_contrastive_bce_loss (plm_emb, labels, model, α, training = True):**

*# plm_emb: protein language model embeddings, shape = (B, seq_len, hidden_dim)*

*# labels: one ‐ hot encoded labels, shape = (B, num_label)*

*# α: weight for contrastive loss, 0.1 in this study*

*# model: sequence/structure/graph model*

preds = model(plm_emb) *# model outputs (logits), shape = (B, num_label)*

**if training**:

# 1. Embed one‐hot labels into same feature space as preds

label_emb = LabelEmbeddingMLP(labels)   *# shape = (B, D)*

# 2. Compute contrastive loss (InfoNCE) between preds and label embeddings

contrastive = NT_Xent(batch_size = B, temperature = T)

C_loss = contrastive(preds, label_emb)   # *scalar*

# 3. Compute standard BCE loss

bce = BCEWithLogitsLoss()

B_loss = bce(preds, labels)   # *scalar*

# 4. Weighted sum

total_loss = B_loss + α * C_loss

**else**:

bce = BCEWithLogitsLoss()

B_loss = bce(preds, labels)   # *scalar*

total_loss = B_loss

**return** total_loss

**end**

A linear layer projects each label representation y into a label embedding *M*, ensuring that *M* has dimensions matching those of the protein embedding *Z*. A mini‐batch of *N* proteins is sampled, producing protein embedding *Z* and corresponding label embedding *M*. The loss function for label embedding alignment is as follows:

(1) $$L_{\text{contrastive}}=-\frac{1}{N\;}\sum_{i=1}^{N}\frac{\log e^{Z_{i}*M_{i}/\tau }}{\sum_{j=1}^{N}\log e^{Z_{i}*M_{j}/\tau^{\prime }}}$$

where τ represents a temperature parameter and * denotes the cosine similarity between the graph and label embeddings of the labeled source data. When i=j, we consider the embedding of protein i, $Z_{i}$ to be correctly matched with its corresponding label embedding $M_{i}$, forming a positive sample pair. Conversely, when i≠j, we assume the embedding of protein i, $Z_{i}$ is incorrectly matched with the label embedding $M_{j}$, forming a negative sample pair.

This loss effectively forces the model to learn representations where positive samples are closer in the high‐dimensional space compared to negative samples. By optimizing this loss, we hypothesize the model learns the alignment between the protein embedding and the label embedding in the latent space, thereby improving downstream label classification. The embeddings from each trained model serve as a foundational input to the contrastive learning framework.

#### 5.1.5 Model training

GOBeacon consists of three distinct models, each trained independently. Details of the hyperparameters including the learning rate, batch size, optimizer, and so forth. during the model training are listed in the Supplementary Material—Methods—Model Training.

#### 5.1.6 Evaluation metrics

To assess the performance of various methods on the PDBch test set, we employ the following primary metrics as per the CAFA (Radivojac et al., 2013) evaluation criteria: (i) protein‐centric *F*_max_ and (ii) function‐centric area under the precision‐recall curve (AUPR). The protein‐centric *F*_max_ calculates the highest F1 score across all prediction thresholds, using a step size of 0.01. The function‐centric AUPR provides a reliable measure for scenarios with significant class imbalances.

#### 5.1.7 Benchmark datasets

##### CAFA3

We conducted an evaluation of GOBeacon using the CAFA3 benchmark (Zhou et al., 2019). The CAFA3 benchmark dataset contains both training and test components. Other leading function prediction models such as Domain‐PFP were also trained on the CAFA3 training dataset (Supplementary Table 1) and assessed using the official evaluation code provided. This training dataset included 66,841 protein sequences that were annotated before September 2016, which encompassed 677 MF, 3992 BP, and 551 CC GO terms. The test set comprised 3328 proteins annotated in the period from September 2016 to February 2017. Following a similar data partitioning strategy as Domain‐PFP, we randomly selected 80% of the training data for model training, reserving the remaining 20% for validation and early stopping.

##### PDBch

To compare with structure‐based function prediction models, we utilized the PDBch dataset used in HEAL and DeepFRI (Gligorijević et al., 2021). This dataset comprises all protein chains from the PDB database for which contact maps were available. Unlike HEAL and DeepFRI, which were both trained on the protein 3D structure, GOBeacon was retrained and tested on the sequences derived from the PDB files from the PDBch training and test sets. These sequences were clustered based on 95% sequence identity, and from this group, representative PDB chains were selected. The selection criteria for inclusion in the PDBch dataset required each protein chain to have at least one functional annotation and a high‐resolution structure. We used the same train‐validation‐test split used in the HEAL paper, in which the dataset was divided into training, validation, and test sets using an 8:1:1 ratio. Specifically, the distribution was as follows: 29,443 sequences in the training set, 3272 in the validation set, and 3414 in the test set (Supplementary Table 2).

Functional annotations were obtained from SIFTS (Dana et al., 2019) and UniProtKB. For a PDB model to inherit annotations, it must exhibit at least 90% sequence identity with, and cover a minimum of 70% of the UniProtKB sequence. Annotations included 489 MF terms, 1943 BP terms, and 320 CC terms, which were used to label each sequence.

#### 5.1.8 Benchmark methods

##### Sequence‐based method

For sequence‐based methods in protein function prediction, we evaluated several models including the Naive approach, which assigns scores based on term frequency in an annotation database. The BLAST (Altschul et al., 1990) method refines predictions by excluding similar sequences and employing BLASTP to find the highest scoring sequence from a training set, adjusting annotations based on sequence identity. Domain‐PFP^12^ uses self‐supervised learning to predict functions by leveraging Domain‐GO associations from protein databases. DeepGOPlus (Kulmanov & Hoehndorf, 2021) combines sequence‐based homology detection with a convolutional neural network, retrained on specific datasets for enhanced prediction accuracy. Details about these methods are included in the Supplementary Material—Method—Benchmark Methods.

##### Structure‐based method

In the realm of structure‐based methods, we explored the HEAL (Gu et al., 2023) and DeepFRI (Gligorijević et al., 2021) models. HEAL constructs a graph input from sequential features and contact maps, using a hierarchical graph Transformer and graph contrastive learning to enhance learning through node feature optimization. DeepFRI integrates protein sequence features and structural insights using a Graph Convolutional Network, trained on both experimental and homology model datasets to predict a broad array of protein functions with high accuracy and residue‐level annotations. Further details on these methods are also available in the Supplementary Material—Method—Benchmark Methods.

## AUTHOR CONTRIBUTIONS

**Weining Lin:** Investigation; writing – original draft; methodology; validation; visualization; writing – review and editing; software; formal analysis; project administration; data curation; conceptualization. **David Miller:** Writing – review and editing. **Zhonghui Gu:** Methodology. **Christine Orengo:** Supervision.

## FUNDING INFORMATION

This work was supported by the Medical Research Council [grant number MR/W006774/1] awarded to DM.

## CONFLICT OF INTEREST STATEMENT

The authors declare no conflicts of interest.

## DATA AND AVAILABILITY STATEMENT

The GOBeacon Python pipeline is freely available on GitHub at https://github.com/wlin16/GOBeacon.git.

## Supporting information


**Data S1.** Supporting Information.

## Data Availability

The data that support the findings of this study are openly available in GOBeacon at https://github.com/wlin16/GOBeacon.
