# Supplementary material for: GOBeacon: An ensemble model for protein function prediction enhanced by contrastive learning
Source: Protein Sci. 2025 Jun 22;34(7):e70182. doi: 10.1002/pro.70182 (PMC12183117; doi:10.1002/pro.70182)
Supplement: Supplementary file 1 — Data S1. Supporting Information. [file PRO-34-e70182-s001.docx]

# Supplementary Material

## Methods

### Sequence-based model

Due to the limitation of computational resources, we had to truncate the protein sequences limiting maximum lengths to 1000 amino acids. For embedding generation, the truncated sequences are fed into the ESM2 model, which features a hidden dimension of 1280. The output embeddings have a shape of batch size, 1000, 1280 and are directly input into an MLP without average pooling of the embeddings.

The MLP consists of two linear layers, the first of which has a hidden dimension of 256. Between these layers, there is a dropout layer at a rate of 0.4 to prevent overfitting, followed by a Sigmoid Linear Unit (SiLU) activation function for non-linear transformation capabilities. Before the final prediction layer, the embeddings are averaged to a shape of batch size, 1, 256. The final output layer is dimensioned according to the number of Gene Ontology (GO) term labels. The final output from the MLP classification output layer is a probability distribution for each GO term.

### Structure-informed model

All sequences were also truncated into the max length of 1000, and fed into the ProstT5 model with the resulting embedding of a hidden dimension size of 1024. Consequently, the output embeddings are structured as [max sequence length of 1000, 1024]. The input then feeds into a simple MLP as in the previous sequence-based model.

### Interaction-based model

Both the GIN and GCN models utilize a two-layer convolutional block setup. In the GCN model, each layer is composed of a convolutional layer, followed by a linear layer, a dropout layer set at 0.4, an activation function (SiLU), and LayerNorm. The sequence ends with global mean pooling to aggregate the learned features from the entire graph.

The GIN model follows the GCN’s layout but replaces the convolutional layer with a graph isomorphism operator, keeping the same hyperparameters consistent.

The GAT model features two layers, each with four attention heads, enabling enhanced feature integration from various viewpoints within the interaction network. The GAT is also configured with a dropout rate of 0.4, an activation function (SiLU), and a LayerNorm layer, concluding similarly with a global mean pooling to combine features from across the graph.

## Model Training

Three models with distinct modalities were trained with the same hyperparameters, include a dropout rate of 0.4 to prevent overfitting and a learning rate of 0.0001. A larger batch size of 512 is used to improve the computational efficiency and stability of gradient estimates. All models are implemented using the PyTorch-Lightning and PyTorch Geometric libraries^1,2^. Although the training process allows for a maximum of 1000 epochs, an early stopping mechanism with a patience of 25 epochs is implemented to prevent overtraining and to halt the training process if no improvement is observed in the validation loss, thereby ensuring that the models do not overfit the training data.

We utilize the Adam optimiser^3^, known for its efficiency in handling sparse gradients and adapting the learning rate for each parameter, facilitating faster convergence. The binary cross entropy loss function is employed to calculate the loss for binary classification tasks (**Equation 1**), measuring the divergence between the predicted probabilities and the actual binary outcomes, making it particularly suitable for models outputting probabilities.

**Equation 1:**

$$L\left( y,\hat{y} \right)=-\frac{1}{N}\sum_{i=1}^{N} y_{i}\log\left( \hat{y_{i}} \right)+\left( 1-y_{i} \right)\log\left( 1-\hat{y_{i}} \right)$$

Where $y$ is the true label, $\hat{y}$ is the predicted label, and $N$ is the number of samples.

Each model undergoes training on a Tesla V100-SXM2 32GB GPU, capable of handling extensive computations efficiently, with total training times nearing 24 hours per model given the extensive batch size and epoch count. This setup not only expedites training but also leverages high-performance computing resources to maximize throughput and efficiency.

## Ablation Tests

### Performance Gains from Contrastive Learning

To evaluate the effectiveness of contrastive learning, we compared models trained with contrastive loss (SSL) and cross-entropy loss (BCE) across CC, MF, and BP categories using paired t-tests.

For CC, the SSL model achieved a significantly higher mean Fmax (0.6800) than BCE (0.6673; BCE - SSL: t = -5.9035, p < 10^-5^). Similarly, in MF, SSL outperformed BCE with mean Fmax scores of 0.6236 vs. 0.6136 (BCE - SSL: t = -3.1102, p = 0.0024). In BP, SSL also showed a significant advantage (0.5713 vs. 0.5619; BCE - SSL: t = -6.311, p < 10^-5^).

These results confirm that contrastive learning consistently outperforms cross-entropy loss across all sub-ontologies. Additionally, contrastive learning may provide efficiency benefits due to its ability to leverage similarity-based training, though further analysis is required to quantify resource savings.

Impact of Graph Information

To assess the effect of incorporating PPI-based graph information, we performed an ablation study comparing ESM2+ProstT5 with and without the GAT module. Paired t-tests were conducted across Cellular Component (CC), Molecular Function (MF), and Biological Process (BP) categories.

For CC, the model with graph information achieved a significantly higher mean Fmax (0.6727) than ESM2+ProstT5 (0.6665; t = 4.4388, p < 10^-5^). In BP, the improvement was also significant (0.5749 vs. 0.5590; t = 13.1941, p < 10^-5^). For MF, the model showed a slight performance increase (t = 1.3602, p = 0.1769), but it was not statistically significant.

These results indicate that while graph-based features enhance performance in CC and BP, their impact on MF is limited. Thus, a dual-ensemble approach using only ESM2 and ProstT5 remains a viable alternative, though the full model benefits from PPI information in certain contexts.

## Benchmark Methods

### Sequence-based method

**Naive**: The Naive approach utilizes term frequency from an existing annotation database as the basis for predicting scores for each input protein. This method straightforwardly assigns prediction scores based on the frequency of terms within the dataset.

**BLAST**^4^: This method involves initially removing sequences from the training set that are similar to the test sequences, employing an E-value threshold of 1e-3. Subsequently, using the BLASTP program, it identifies the sequence from the PDBch training set with the highest score. The annotations of this sequence are then adjusted based on sequence identity to the query sequence to determine the predicted annotations.

**Domain-PFP**^5^: Domain-PFP leverages a self-supervised learning approach to predict protein functions by exploiting the domain-GO associations discerned from protein databases. This method embraces the co-occurrence of domain and GO terms across a large dataset to generate pseudo-labels for GO prediction probabilities. Subsequently, it employs these labels to craft a dense representation of domains aligned with functional information, which is then utilised to predict protein functions.

**DeepGOPlus**^6^: This hybrid method combines sequence-based homology detection using DIAMOND Blast with a one dimensional convolutional neural network. For our evaluation, DeepGOPlus was retrained on the PDBch and SMch datasets, with an adjusted weight combining the DIAMOND Blast score and the neural network score, refined based on results from PDBch and SMch validation sets.

**PhiGnet**^7^: This sequence-based method utilizes statistics-informed graph convolutional networks, integrating evolutionary couplings (EVCs) and residue communities (RCs) derived from multiple sequence alignments to predict protein functions. PhiGnet applies a dual-channel graph convolutional architecture to learn functional representations from evolutionary data, without requiring explicit three-dimensional structural information.

**MIF2GO**^8^: This multimodal method integrates heterogeneous biological data across six multiple modalities, including protein sequence, domain annotations, protein-protein interaction networks, homology relationships, subcellular localization, and pathway information. Specifically, MIF2GO employs a Siamese Contrastive Autoencoder (SCA) to fuse domain, localization, and pathway modalities with interaction and homology data through self-supervised learning, followed by a Language Model with Hierarchical Adaptive Weighting (LM-HAW) to capture hierarchical features from protein sequences. Finally, these multimodal embeddings are combined using a Modal Hypernode Pooling (MHP) layer, resulting in unified protein representations that significantly enhance protein function prediction.

### Structure-based method

**HEAL**^9^: The Hierarchical graph transformEr with contrAstive Learning (HEAL) constructs a graph input combining sequential features and contact maps. It employs message passing neural networks for gathering short-distance information and a hierarchical graph Transformer to explore and aggregate long-distance node correlations adaptively. This approach also introduces graph contrastive learning to enhance model learning by optimizing node feature perturbations and similarity scores across different views.

**DeepFRI**^10^: DeepFRI combines protein sequence features derived from a protein language model with structural insights using a Graph Convolutional Network (GCN). This method is trained on datasets from PDB and SWISS-MODEL, leveraging both experimental structures and homology models to predict a broader array of protein functions. By capturing complex interactions within protein structures, DeepFRI achieves high accuracy in function prediction and allows for residue-level function annotations.

**DPFunc**^11^: This deep learning-based method integrates domain-guided structural information to accurately predict protein functions. DPFunc leverages residue-level features extracted by a pre-trained protein language model, combined with structural relationships learned through Graph Convolutional Networks (GCNs). Crucially, it utilizes domain annotations obtained from protein sequences to guide an attention mechanism, effectively identifying functionally important residues and structural motifs. This approach significantly enhances interpretability and accuracy, achieving state-of-the-art performance in structure-based function prediction tasks.

**Supplementary Table 1**: Overview of CAFA3 Benchmark Dataset

| Ontology | Number of GO terms | Number of Training set | Number of Test set |
| --- | --- | --- | --- |
| MF | 677 | 36,110 | 1,137 |
| BP | 3,992 | 53,500 | 2,392 |
| CC | 551 | 50,596 | 1,265 |

**Supplementary Table 2**: Overview of PDBch Benchmark Dataset

| Ontology | Number of GO terms | Number of Training set | Number of Test set |
| --- | --- | --- | --- |
| MF | 489 | 24,604 | 3,414 |
| BP | 1,943 | 23,216 | 3,414 |
| CC | 320 | 11,174 | 3,414 |

# Reference:

1. Fey M, Lenssen JE (2019) Fast Graph Representation Learning with PyTorch Geometric. Available from: http://arxiv.org/abs/1903.02428

2. Falcon W (2019) PyTorch Lightning. Available from: 10.5281/zenodo.3828935

3. Kingma DP, Ba J (2017) Adam: A Method for Stochastic Optimization. Available from: http://arxiv.org/abs/1412.6980

4. Altschul SF, Gish W, Miller W, Myers EW, Lipman DJ (1990) Basic local alignment search tool. Journal of Molecular Biology 215:403–410.

5. Ibtehaz N, Kagaya Y, Kihara D (2023) Domain-PFP allows protein function prediction using function-aware domain embedding representations. Commun Biol 6:1–14.

6. Kulmanov M, Hoehndorf R (2021) DeepGOPlus: improved protein function prediction from sequence. Bioinformatics 37:1187.

7. Jang YJ, Qin Q-Q, Huang S-Y, Peter ATJ, Ding X-M, Kornmann B (2024) Accurate prediction of protein function using statistics-informed graph networks. Nat Commun 15:6601.

8. Ma W, Bi X, Jiang H, Wei Z, Zhang S (2024) Annotating protein functions via fusing multiple biological modalities. Commun Biol 7:1–13.

9. Gu Z, Luo X, Chen J, Deng M, Lai L (2023) Hierarchical graph transformer with contrastive learning for protein function prediction. Bioinformatics 39:btad410.

10. Gligorijević V, Renfrew PD, Kosciolek T, Leman JK, Berenberg D, Vatanen T, Chandler C, Taylor BC, Fisk IM, Vlamakis H, et al. (2021) Structure-based protein function prediction using graph convolutional networks. Nat Commun 12:3168.

11. Wang W, Shuai Y, Zeng M, Fan W, Li M (2025) DPFunc: accurately predicting protein function via deep learning with domain-guided structure information. Nat Commun 16:70.
